# Report of the Mississippi Val. Association

**Published:** 1858-04

**Authors:** 


### FOURTEENTH ANNUAL MEETING OF THE MISS. VALLEY ASSOCIATION OF DENTAL SURGEONS.

### DISCUSSIONS.

1. *What means will be most efficient in securing a healthy denture?*

Dr. Taylor remarked, that he would merely rise to open the question—would not probably occupy the ten minutes allowed. He remarked that the question was important, as retaining a tooth in a healthy state, is more important than restoring it by an operation, or replacing it by a substitute. He would not consider the causes of caries. The condition of the tooth predisposing to disease is often established in early life—too early for professional interference. Indeed, it would require us to begin as early as the first connexion of the sexes. But we can not control this. People will marry; and young men seldom think of the dental organs when in love. The first evidence of provision for the dental organs is manifested about the sixth week of fœtal life. If we could then introduce, into the fœtal system, all the elements necessary to a perfect development of the teeth, much would be accomplished. But how can we control this? To accomplish any thing, we must begin with the mother. That mother who pays but little attention to her own health is culpably careless of that of her offspring. We find elements in the blood from which the organs are developed. The health of the blood is, therefore, an important consideration. The crowns are already formed at birth. The maternal influence previous to this is beyond our reach, unless we have direct control of the mother. Mothers do not understand this.

Dr. Taft said that this question is of great moment—is the *first* question for our consideration, and very properly stands first in our programme. It is difficult to find a practicable starting point. We must, as has been remarked, begin with the parent. The most perfect condition of health should be secured to the mother, even before conception. We can not tell how far the influence of the parent will be communicated to the child. The offspring sometimes inherits the peculiarities of but one of the parents,—sometimes of both. If the constitution of the child be better than that of the mother, the question arises, what can be done in the way of direct nourishment.

The mother's health must be kept as perfect as possible during gestation. Undue excitement of mind or body should be avoided, for the child will be influenced, even by the disposition of the mother during gestation.

There is often a deficiency of ossific matter in the system. The mother's milk should be of the proper kind and quantity. Her system must appropriate the materials necessary to development of the organs. She should, therefore, have proper food, and should remember that her mental state modifies digestion. Many do not know these things; we should, therefore, endeavor to enlighten the minds of those with whom we are associated. The mother's direct influence continues after birth, through the medium of lactation. The mother's milk often affords but imperfect nourishment. The constitution of the child must then suffer, even if it be able to assimilate properly. The same general course, in regard to the health of the mother, as described above, should still be pursued. The health of the child in early life has much to do with the condition of the dental organs, as, for instance, atrophy is, doubtless, the result of interrupted development. The health of the child must be properly cared for, both before and after lactation.

Dr. Blandy remarked that it was proper to consider how we can control the general health. What is healthy to one constitution is unhealthy to another. We can only recur to the hygienic laws which experience has defined to be healthy. The times of eating should be regular. It is useless, if any ingredient is deficient in the system, to prescribe food containing it. He would advise nursing till the primary teeth are erupted. He advises cleanliness of the child's mouth, even before the teeth appear. He believed we could, in a measure, control the child's constitution, even if the parents be unhealthy. He was a great advocate of filing and filling the temporary teeth.

Dr. Watt said that the constitution of the parents had much to do with that of the offspring. Though morbid conditions are more likely to be inherited from the mother than from the father, yet the influence of the latter was very decided. We often see the child inherit the exact dental peculiarities of the father. But as parental influence has been already insisted on, he would direct attention to the care of the child after the period of nursing. He would remark, in the out set, that if any element essential to health be deficient in the system, it is important to prescribe food containing it, as the laws of life never violate the principles of chemistry, and the nutritive functions can not create, but only assimilate materials furnished for them to act on. As the condition of the deciduous teeth influences materially the health of the permanent ones, they should receive early and close attention. We should endeavor to obviate the tendency to decay, whatever that tendency may be. For example, if we find the usual form of "white decay" making its appearance, we are warranted to infer that nitric acid is the destructive agent, and accordingly, we should, if practicable, limit the quantity of nitrogenous food, and especially we should prevent the putrification of particles of animal food or mucus about the teeth. If the tendency be to that form of decay in which the animal portion of the tooth remains while the calcareous matter is dissolved out, we must endeavor to lesson the use of food highly seasoned with common salt, (chloride of sodium), as hydrochloric acid is the destructive agent in such cases. That condition called "chemical abrasion" being usually caused by lactic acid, if it appears, we should prohibit the free use of such food as yields this acid by fermentation. The principle is to correct the morbid tendencies as soon as they manifest themselves.

Dr. Taylor remarked that when up before, he was like a young lover before his avowal—he hadn't much to say—now he feared he would incline to say too much. This discussion reminded us of the importance of chemical science, of which we had just enough to lead us to desire more—a few data, now and then a fact, from which we can reason.

Peculiar characteristics are transmitted from generation to generation, and the dental organs are, by no means, an exception to the rule. There is, if you please, a germ force which we can not control; but we may modify and change the material, if not the form of the organs. It is true the mother may not always be able to digest the materials which her system requires, for the elaboration of these organs. That which would give the best dental organs might not give the best muscular development. If the mother be feeble we can not control the child till birth; but after this we can supply it with a healthy nurse. The French and English understand this matter better than we do. It is understood that the kind of food used has a direct effect on the constitution of the person using it. One fact is important to be remembered. The tooth is not perfect as soon as it is formed. Till adult life it still receives matter, to enable it to resist active corrosive agents. And diseases which alter the blood prevent the natural hardening of the dentine. Even farther; the progress of caries is much facilitated by the condition of dentine thus developed.

In relation to diet, we find one member of a family craving one kind of food, and another a different kind. This *may* indicate the wants of the system; but we must watch the tendencies. The sanguine temperament gives usually the best constitutions, yet in this temperament we often find defective teeth. This may arise from the influence of acute diseases, to which such persons are especially liable, taking place during the development of the teeth. Bad teeth in a good constitution, often indicate that the constitution was deficient during dentition, but has improved.

Dr. Webster inquired if peculiar conditions of the teeth would sometimes indicate the family to which an individual belonged. He reported a case in which a lateral incisor decayed early, in a whole family.

Dr. Bonsall said there was no question that we may improve the state of the permanent organs by the use of food containing the ingredients of tooth bone. He urged the best possible care of temporary teeth till the permanent ones take their place.

Dr. TAFT said that was an important idea mentioned by Dr. Taylor, that the tooth continues to solidify as life advances. This should be promoted. The pulp cavity is gradually filled up. This secondary formation should be perfectly formed.

Now, as to the practicability of counselling the mother there is no difficulty. The most fastidious will listen, with pleasure, to your advice, if she is assured you possess valuable knowledge on the subject. He spoke of subphosphate of lime as the ingredient usually defective in badly developed teeth, and gave a number of cases in which its administration had been followed by highly satisfactory results.

2. *What is the most effectual treatment of exposed nerves; and what circumstances modify the treatment?*

Dr. TAFT supposed it would be best to consider the treatment most efficient in ordinary cases. If the pulp was exposed at merely a small point, and was healthy, he would place a non-conductor over it and fill as in ordinary cavities. As non-conductors he used oil-silk, or gutta percha, coating the under side of the silk slightly with adhesive wax, or a similar preparation, to retain it in place. If the exposure be considerable, he would often proceed on the same general principles. He would be very cautious in introducing the gold. Would begin the filling at one side of the cavity, and then at the other, never make direct pressure over the exposed nerve. Thus he would arch over the exposed point, and then fill up the cavity. In answer to a question, he said the non-conductor should lie right on the nerve.

If the exposure be considerable, and the pulp unhealthy, the treatment must be modified by circumstances. The previous constitution of the patient, the present state of the health, and the condition of the pulp, must all be carefully considered. Constitutional treatment may be required in some cases, in others, local means will answer. The pulp must sometimes be depleted; as when it is in the main, healthy but acutely inflamed. He would, in such cases, puncture it with a fine pointed instrument. Depletion of the gums is often serviceable; and local astringents or escharotics may be often used with advantage. In some cases he would make no attempt to save the pulp alive.

Dr. BLAKESLEY—How many teeth do you save by this treatment?

Dr. TAFT—More than two-thirds of the number treated; but I select the cases. If tried indiscriminately, a large majority will fail.

Dr. BLAKESLEY said he expected they would. He seldom treated nerves, or succeeded in saving them. He could not say much; as he treated exposed nerves as open enemies—waged on them a war of extermination. He presumed there must be a modifying influence in climate, and believed that the treatment detailed above would not suit his locality.

Dr. Blandy remarked that he never allowed his non-conductor to touch the nerve. It was a foreign substance, and he would consider it a moral impossibility to save a nerve alive with such an irritant in contact with it. He did not think an arch could be built as described over an exposed nerve. His method was to choke up the cavity and case harden the surface with a fine pointed instrument.

Dr. Taft spoke of his want of success in, and his objections to leaving a vacant space over the exposed point.

Dr. Blandy replied, at some length, in favor of the mode, and objected to the mode adopted by Dr. T. (The remarks of both gentlemen at this point, we failed to get accurately.)

Dr. Taylor remarked, that in looking back over his practice, he could not tell when he began the operation of capping. When he was young, it was seldom attempted; yet, even then, the profession were anxious to save the teeth without destroying the nerves. He described the old method of capping, the object of which was to avoid pressure on the pulp, but, the confined air and the fluids exhaled produced pressure and thus defeated the effort. For the last four or five years he had followed the plan just laid down by Dr. Taft. He wanted neither pressure nor a vacuum. In some cases no attempt should be made to save the pulps. Tooth-ache is easily induced in some constitutions, in others it is not. In the latter class he would endeavor to save, in the former he would not. He would not condemn a tooth because the pulp had been inflamed. Why, said he, can we not sometimes subdue inflammation here as in other places? He does not always use a non-conductor—always does if cold causes pain. Uses oil-silk, Hill's stopping, or a similar material, the object being to get an indestructible non-conductor. It was once supposed that if a pulp was wounded it was lost. Now he sometimes wounded it to deplete it.

Dr. Ulrey believed that it was generally the best plan to destroy exposed nerves; but the question being on *treatment*, he mainly coincided with the remarks just made. When the nerve was irritated he generally used creosote or chloroform as a local application.

Dr. Webster would like to have a general expression from the members. He generally failed with treatment, and usually extirpated the nerve.

Dr. Taylor remarked in explanation, that he does not cap as frequently as formerly, but makes it a point, if possible, to save the nerves in front teeth.

Dr. H. R. SMITH remarked, that he tries a little of almost every thing. He had doubts about the success of modes in which others professed to succeed; as in bleeding the nerve, &c. If he wounds the nerve, he dont fill until he treats. He often uses a solution of tannin in glycerine. If the pulp has ever been highly inflamed he would not attempt to fill over it. He was not skillful enough to arch over an exposed pulp, or to fill satisfactorily over a non-conductor. This puncturing the nerve is but a modification of the Hullihen operation, which all had abandoned. Puncturing the nerve is pretty in theory, but he could not work unless he could see. He did not believe in the existence of healthy teeth with dead nerves. The tooth is, then, he maintained, a foreign substance. He gave instances of unsuccessful cases in his own practice and in the practice of others. He said that nature does sometimes tolerate the teeth in which he kills the nerves. He wished to report his unsuccessful cases.

Dr. TAFT explained, that when the pulp is exposed at but a small point, in a good constitution, he either filled at the first sitting, or temporarily closed up the cavity. In the latter method the pulp often throws out ossific matter and protects itself. He had several cases now on hand. He had no doubt that the same result often follows permanent fillings. When decay approaches the pulp, he had no doubt that deposits of osseous matter took place more rapidly than at other times. He remarked that he usually treats from twenty to thirty cases of exposed pulps, in the manner described, every year, keeps a record of the cases, and *knows* that many are successful. An objection to the unnecessary removal of the pulp, is, that a dead tooth decays more readily than a living one, and is more liable periostitis. It is better, therefore, to retain the pulp, if practicable. The tooth is not wholly a foreign or dead substance when the pulp is destroyed. He has noted his cases of exposed pulp for many years, and claims to know whether he has succeeded or not. He would not disbelieve those who say they fail in the use of the same means, but only claimed a right to be believed himself.

DR. BLAKESLY said he believed every word, and would go home and act on the suggestions presented. He had succeeded badly by this mode, and so much better by extirpation, that he had almost exclusively adopted it.

Dr. CHAPMAN, (physician) supposed the question involved the subject of inflammation. He would call attention to inflammation, as it is modified by texture. This idea was not noticed at an early day, with sufficient care. For example, rheumatism is very painful, the physical structure of the part involved being the cause. He thought the same principles might apply to the textures of the tooth. The progress of inflammation is much modified by texture. In serous tissues its progress is rapid, in mucous structures, slow, in muscular, intermediate, and in the bony, very slow. The termination and duration are both materially modified by texture. He would like to ask how long patients with inflamed pulps had been subjected to treatment. He said that the intense pain resulting from this disease, arises from the pressure incident to expansion of the pulp in its bony canal, and thought prompt relief could be given by but two methods, extraction and extirpation. He inquired if it was practicable to subject the patient to such severe pain; and if so, how long? A longer time would be required than with inflammation of the soft textures.

Dr. TAYLOR remarked, that great wisdom is displayed in the formation of all parts of the system, and in none is this more evident than in the dental organs. If the teeth were constituted as other bones, a loss of vitality in one part would result in its exfoliation. The crown, deriving its nutriment from the pulp, necessarily dies when this is extirpated, yet the vitality of the crusta petrosa is not sufficiently active to cause its exfoliation. He said the treatment of exposed nerves was not like that of inflamed dentine. The latter often requires a longer time than the former. If a nerve be exposed, and free from inflammation, nature may, and often does, protect it by a bony deposit, if irritating agents be excluded. If the pulp be wounded, it heals by first or second intention. If by the latter, pus is formed, and pressure would result, if the tooth were filled without treatment. He considered the life of the pulp essential to the vitality and natural color of the crown. He only attempted to save exposed pulps in favorable cases.

Dr. TAFT called attention to the fact that the tooth-pulp is a soft texture, and is, therefore, amenable to rapid treatment.

Dr. BLANDY would like to have the gentleman define inflammation.

Dr. CHAPMAN, in reply to Dr. B., described the pathological conditions constituting inflammation. He also remarked, that it was sometimes difficult to determine the existing stage of inflammation. He said that disease often began before pain was manifested. In confirmation of this, he exhibited a tooth, with extensive absorption (abrasion?) of the fangs. The extent of the absorption was evidence that the disease had existed a considerable time, yet the pain was quite recent. He only intended to suggest points for examination.

Dr. WATT remarked, that he had great confidence in the treatment of exposed pulps when the cases were judiciously selected. In general his treatment was similar to that described by Dr. Taft. A healthy pulp, in a good constitution, is not always destroyed. He referred to a case in which arsenious acid was applied directly to the pulp, four different times, the access being easy and the pulp in full view, yet its vitality was not destroyed. A change of treatment was adopted, and when the pulp became healthy, it was protected by a non-conductor, and the tooth was filled. A year and a half afterwards, it had given no pain, and still retained its natural color. If the constitution be good, and the pulp healthy and but slightly exposed, he would be surprised if, when properly protected, it would fail to shield itself by an ossific deposit, just as he would be surprised if a good constitution should fail to effect a bony union in simple fracture.

3. *Filling Teeth.*

Dr. BLAKESLEY remarked, that in filling teeth he uses crystal foil. He tried crystal gold; but this was not always satisfactory, as it lacked uniformity. Some of it was very good, and other specimens were not. He uses the crystal foil, because it is chemically pure gold, and is remarkably adhesive. He thought its superiority over other foils in this respect, was owing to its greater purity. In very deep cavities he uses foil in its ordinary condition, till the cavity is nearly full, and then finishes with adhesive foil. Adhesive foil, if properly introduced and condensed, becomes a solid mass. He makes, what he calls a complicated cavity—small pits or grooves being formed in the walls of the cavity, to prevent the displacement of the filling. By this mode he can fill out from the margin of the cavity to restore a disfigured tooth to its original form. This mode might be considered tedious; but, since he had adopted it, he had trebled his prices, and doubled his business. In the front teeth he forms the cavities with small broaches, making depressions as starting points, cutting away a little of the healthy bone to obtain the desired angle. He then makes a dowel-shaped space next to the cutting edge of the tooth. (He explained his excavators and the form of cavities, which he preferred, very minutely, but we can not do justice to this part of his remarks.)

The cavity being ready and the instruments selected, he takes Watts' crystal foil, No. 4, and rolls a sheet into a rope, and cuts it into pellets, with their length and thickness about equal. He begins with the largest; and if it do not lie steady, he thrusts an instrument between the gold and the wall of the cavity and fills the space thus made with small pellets. When desirable, he builds out and restores the form of the tooth. He had adopted this method within the last year; and, as regards this, he was willing to be considered either a young man, or an old boy. In the treatment of fangs he removes the nerve with a plain broach, not too small, and the temper should always be drawn before a broach is used for this purpose. He uses linen fibre to dry the canal. He remarked that he does not fill the canal with wire; indeed it was sometimes too small to be filled by any mode. He forms his foil into pointed cylinders to introduce it. If an abscess be present, he prefers to have it healed before filling the fang. He said that but slight discoloration was caused by the death of the nerve. He had great faith in fang filling—had treated a great number of cases, without any failures known or reported.

Dr. TAFT said that he would confine his remarks mainly to fang filling. He wanted a direct entrance into the canal. This can not always be obtained through the cavity of decay; as in a proximal decay of an incisor, near the neck of the tooth. Then he would drill through the palatal surface of the tooth, in a line with the canal. Then he sometimes takes a tapered broach, inserts it and rotates it till the canal is perfectly cylindrical. Then he would prepare a gold wire, the size and shape of the broach, and insert it. But sometimes he fills with foil. This is necessary when the entrance is not direct. In young persons, the instrument or the gold may project through the canal. There are two modes of preventing this. One is to introduce a little pellet as far as necessary, measuring the distance, and let the ends of the cylinders rest on this. The insertion of the wire has sometimes an advantage not mentioned before. In a badly shaped, frail cavity, it may be allowed to project, and, in filling the cavity, the gold may be condensed around it.

Dr. I. FORBES described his mode of filling a compound cavity in a bicuspid; but for want of a definite diagram, we failed to understand him with sufficient clearness to do him justice. The same is true of Dr. Blakesley on the same cavity.

Dr. TAYLOR described his mode of filling the same cavity. He also reported a case of exposed nerve which he destroyed five days ago. He had since filled the palatal fang—was not satisfied that the gold had reached far enough. Periostitis followed, and to-day he extracted the tooth, which exhibited extensive absorption of the roots. He wished to know if this could take place, to such an extent in five days, and would like to know if abscess may be developed while the nerve is still alive.

Dr. HUNTER described, by diagram on the blackboard, his mode of filling a proximal cavity in a bicuspid. He makes pits or depressions at the parts of the cavity next to the neck of the tooth, and next to its grinding surface. These pits he fills with soft (unannealed) foil, and he finishes with annealed foil. He lets the soft foil represent the dentine, and the annealed, the enamel.

Some superior fillings, both in and out of the mouth, were exhibited; and a free exchange of ideas in regard to them prevailed, without formality or adherence to parliamentary rules. All seemed to enjoy the exhibition.

Dr. TAYLOR maintained that the best mode of filling is that which will accomplish a good operation in the least time. Ease to the patient and the necessity for dryness were important considerations. He prepared both the cavity and the gold for the quickest effort, and frequently was not more than two or three minutes in introducing the gold. The present improvement in foil. he said, somewhat obviated the necessity for blocks. Our old *strip* fillings would fritter away, as they did not adhere. The blocks obviated this difficulty. Now, however, we can, on account of the adhesiveness of the gold, begin at the bottom of the cavity. He began to use blocks, in filling, as so many wedges. He can fill ordinary cavities with blocks quicker than by any other method, except amalgam. He likes adhesive gold, but does not always use it. He wants his foil to adhere, block to block. There should be no sudden curves or projections in the cavity. The gold should be rolled firm; and the first block should be as large as will readily enter the cavity. Then force in a condensing instrument and press to the bottom, and finish at the point least likely to get wet.

But if he wishes to build out from the cavity, then he must have adhesive gold. He inquired to what extent we should carry this building out. Not how far can we, but how far *should* we? As an illustration of the adhesiveness of gold, he alluded to crystal gold, and to a ring made from foil, by Dr. Leslie, which, after a year's wear was as firm as ever. He referred to fancy operations described at length by a friend in the east, and inquired as to their practicability. How many would pay for them? and how long would they last? He did not disapprove of efforts in this direction, but thought the building out process not usually the most useful. For example, when the inner cusp of a bicuspid is destroyed, if in filling, the tooth were converted into a *cuspid,* he maintained that it would last longer than if the inner cusp were built up with gold. He would not of choice build out a corner to a front tooth. The space would be less offensive to the eye than the gold. A molar should be built up when an antagonizing point is needed to protect an artificial denture.

Dr. BLAKESLY would not be understood as an advocate of extreme outbuilding, especially in front teeth. He only spoke of it as something that can be now done, but could not formerly. He remarked that much taste might be displayed in the separation of the teeth. Filling with blocks, he remarked, takes less time. He will try it for a great many cavities. He doubted the welding of the blocks, but in many cavities, he said, it would make no difference. If the cavity is badly shaped, he would prefer the adhesive foil. He proposed to experiment with Dr. Taylor's mode, if Dr. T. would with his.

Dr. TAYLOR agreed to the proposition, and remarked that he had been using adhesive gold to fill up parts of cavities where his blocks would not occupy the entire space without bending.

Dr. LESLIE proposed to make a few remarks on materials for filling, and would say all that he could in favor of Watts & Co's foil. He stated that there was none in market more adhesive. He thought much would be known, a year from this in regard to foil that is not known now. Watts & Co. he thought were convinced that crystal gold was not the thing; and the profession are being convinced that foil is preferable. He would leave the profession to decide on the merits of W. & Co's gold. Others *might* be able to do what they can. All chemical knowledge was not vested in Watts. He thought it was not best to speak so positively in recommending our wares. Fallible men should allow themselves room to improve. He kept W. & Co's foil for sale. He was not here as an advocate of Leslie's foil.

Dr. BLAKESLY claimed that Watts only can make pure gold.

Dr. WATT remarked that such a claim was calculated to injure the interests of Watts & Co., for, as it was not well founded, its tendency was to excite prejudice against their gold, as most persons instinctively conclude that a good article needs no such claim to support it. The claim was also unjust to other manufacturers, and to chemists in general. He had tested several varieties of foil, among them that of W. & Co., and had found them pure gold. He liked the Utica foil, but not the ungenerous claim by which it is endeavored to introduce it. As a friend of A. J. Watts & Co., he regretted to see such a claim set up for them, and hoped it would not be pressed to their injury.

4. *What are the indications for the extraction of deciduous teeth?*

Dr. ULREY remarked, that uncontrollable pain, the age of the patient, the appearance of the permanent organs, abscess, if suppuration be extensive, and especially if accompanied by ulceration, were the principal indications for the extraction of the temporary organs. He said it was very important to palliate, in many cases, on account of the irregularity of the permanent teeth, which was likely to result from the early extraction of the deciduous organs; but sometimes early extraction is necessary. He reported a case, in which he had extracted the lower centrals of a child, five months old, on account of the injury which they inflicted on the tongue while the child was nursing. The tongue was badly cut, and much swollen, and the child suffered severe prostration from want of nourishment. He endeavored to shield the tongue by capping the teeth with wax, &c., but failed, perhaps for want of perseverance on the part of the parents; and, as the child was rapidly failing, he extracted the teeth. The patient is now about ten years old, and the permanent centrals have not yet appeared.

He would rather have the deciduous teeth remain too long than removed too early. It is better to submit to a little toothache. The contraction of the jaw, or rather the want of expansion, is the usual consequence of too early extraction.

Dr. BONSALL desired to impress on the minds of young members to be cautious when solicited to remove the deciduous teeth on account of pain. In a large majority of cases in his own practice he refused to extract them. They should remain till the permanent teeth appear, unless the pain be unbearable and uncontrollable.

Dr. STIPHER said his views were, in the main, expressed by Dr. Ulrey.

Dr. KING reported a case in which he removed an upper tooth for a child three months old, for the same reason as in the case reported by Dr. Ulrey.

Dr. TAFT remarked that he would always try to cure the tooth-ache; and even periostitis can mostly be managed. If an abscess be formed, the germ of the permanent organ would most likely be destroyed. He mostly used, as an application to the pulp, in the toothache of children, a mixture of alum and sulphuric ether triturated together. Saline purgatives would be beneficial in both this and periostitis. At the proper age for shedding, if the permanent teeth were pressing in a wrong direction, he would remove the temporary, even if they were not loosened. They must be carefully removed in such cases, or the germs of the permanent organs will be injured.

Dr. RICHARDSON remarked, that he was often at a loss whether to remove all the incisors, or only the central, when the permanent centrals are too large for the space of the two temporary teeth.

Dr. BONSALL said, if the permanent occupied the space of the temporary centrals, and half that of the laterals, he would only extract the centrals. Sometimes, however, he considered it necessary to remove the four incisors, and, finally a bicuspid or first molar, and then regulate.

Dr. FORBES never extracts more than one tooth to make room for another. The expansion of the jaw is continual. The roots are smaller than the crowns. The sides of the fangs are abraded to make room, if the permanent organs mis-shoot. The under jaw is the great trouble in irregularity, and the difficulty is growing rapidly worse. When asked why, he jocularly remarked, that people don't drink as good whisky as they formerly did; or, in other words, the laws of life are more frequently and flagrantly violated.

Dr. WEBSTER referred to the death of the tooth, and the exposure of the fangs by absorption and ulceration of the process and gum, as an indication for extraction.

7. *What are the merits and demerits of that method of inserting artificial teeth denominated "Cheoplasty?"*

Dr. BLANDY said, it was not from choice that he would discuss this subject. The dental profession is essentially and *peculiarly* practical, and must do with results rather than theories. He would rather his method was submitted to practical test, instead of theoretical discussson. Theorists are often much sought after, while practical men are neglected or persecuted. Mesmer and Hahnemann aroused the credulity of the masses, and were thus aggrandized, while the inventor of gas died in jail.

He advised caution in regard to the habit of condemning before trial. He asked the profession to look in moderation on this mode. It was highly endorsed by men every way competent to judge of it. He had used it successfully himself for two years and a half. He would not have been here had not this been adopted as a subject for discussion. This style of work, he said, required nicety of manipulation, and is applicable to all cases. These specimens are all duplicates of practicable work. (Dr. B. here exhibited a great number and variety of pieces of Cheoplastic work, W.) He challenged the profession to find a case in which he can not insert with advantage. Patients, often, without difficulty, eat the first meal after the insertion of a single tooth, without in the least displacing it. He did not know of a single case in which this work is not successful, except a few cases which he had recently made in Cincinnati. If the model is correct, a perfect fit may be obtained. Chemically speaking, he said, his metal might shrink; and shrinkage might vary. He claimed that his *compound* is a *single* metal. The elements composing it may be—they are all objectionable; yet, said he, their combination is, according to equivalents, a new, pure metal. Tin, he said, would shrink, and is too soft; and its contact with the platinum pins would destroy one or other of the metals. You know the bad effect of silver, and of antimony. You know the powers of gold—that it is totally changed by another metal. Many phenomena arise from the combination of metals; and these phenomena must be admitted, but they can not be explained. I know some pretend to; but there is such a thing in our profession as chemistry run mad. The mouth, he maintained, would sometimes act on gold and platinum; but it was not free acids that produced the action, nor sulphuretted hydrogen. He denied that *free* acids are ever found in the fluids of the mouth. He stated that he knew his metal was practically stronger than gold, and again challenged the profession to give him a case he could not mount. This style of work he considered better adapted to broken than to full sets, and was as pure to the mouth as ordinary gold plate.

Dr. B. then requested any member, who desired farther light on the subject, to suggest any inquiry he saw proper.

Dr. RICHARDSON. Can you get a uniform metal?

Dr. BLANDY. I believe I can, mechanically; I would not speak of exact chemical analysis. I test its specific gravity, shrinkage, fusing point, &c. I don't profess to know much about chemistry. As to weight, he believed he could make a lighter job than could be made by any other metal. These metals have certain elementary powers of combination which induce them to unite in the proper proportions to form a pure metal.

Dr. BLAKESLY. Is it applicable when it is desired to restore the features?

Dr. BLANDY. It is better than anything else, and is more easily and speedily applied.

Dr. H. R. SMITH. Will plating last on it?

Dr. BLANDY. Don't plate it. Plate it as you will, you will have apertures; and the result is galvanic action.

Dr. TAFT remarked that he had put up six or eight cases—didn't know whether any of them are now worn or not. An upper set was worn two months, and was not corroded to any extent, and no taste was complained of by the the patient, who, however, was not satisfied with the job. She now has a gold one, and is not as well satisfied with it.

D.r TAYLOR knew but little of this work, personally, till within the last ten days. He reported a case in which Drs. Harris and Blandy, and afterward himself, had failed to get a good fit with gold, Dr. Blandy had succeeded in getting a good fit by his method, within the last few days. Another case a good fit, worn five days and not discolored. He had no doubt about the fit, has less than formerly on its strength, and would not object strongly to the weight. He expects to use it in many cases. It does not tarnish as much as silver, and takes but little time.

Dr. BLANDY stated that it was not more difficult than other work, was accurate and took less time. He described the mode at some length; for which the reader can consult his circulars.

6. *To what extent may the atmospheric principal be applied to partial sets of teeth?*

Dr. H. R. SMITH had not been very successful in mounting single teeth, on this principal, except when the plate was very large. Most patients were not willing to pay prices to justify. He thought most cases could be made to articulate, and be useful in mastication, by making the plates large enough. How they can be made to masticate on a plate the size of a dime he could not see. In reply to a question from the president, he said he liked the central cavity, except in deep arches. Strangulation of the mucous membrane is sometimes produced. His object in constructing the chamber is to avoid pressure on the roof of the mouth.

Dr. BLANDY said, we had better look with care on this subject, as the central cavity is in almost universal use. A properly constructed chamber gives the patient a control of the work, "a volition," and he considered it a necessity in partial sets, unless they are clasped. It is only the abuse of the cavity that is injurious.

Dr. BONSALL doubted that the chamber, without a valve, becomes a vacuum, or renders the plate more adhesive.

Dr. WEBSTER thought all would agree that the cavity plate sticks better at the start, he wished to know if it continued to do so.

Dr. BLANDY said, that the mouth varies in size, and the cavity permits the patient to have a voluntary power over the work.

Dr. SMITH had a different idea of atmospheric plates. He believed that chambers had come up to atone for bad fits. If the fit be perfect, the pressure is nearly fifteen pounds to the inch. If a plain plate be a perfect fit, it would stick tighter at the start than one with a cavity. The cavity has its uses—it is fashionable and it stiffens the plate. An objection to it is that it spoils the contour of the mouth. When used, it should be so placed as to prevent the rocking of the plate on the median line.

Dr. TAFT said he had used chambers, but thought he would return to plain plates. Five years ago, he made nearly all plain. But, to get at the question, he thought it very desirable to adopt this mode in partial sets, when practicable. In what may be called "ragged jobs," a tooth to be replaced, here and there, he thought it mostly practicable. With bicuspids and canine of the same side gone, it would be difficult to replace them by this mode, especially if the arch be deep.

Dr. KING had not succeeded very well with partial sets by this method.

Dr. VANEMON was governed mostly by the remaining teeth. If they were not liable to be injured by clasps, he would not use suction, otherwise he would. He did not consider cavities of much use. He placed a sheet of lead, or something similar, under the plate and struck it up.

Dr. KNOWLTON was often annoyed by the prominences on the cast being bruised down in swagging. He relieves it by sheets of lead under the plate, as alluded to by Dr. Vanemon. He thought that adhesive power was gained by the chamber. The median line was apt to defeat a plain plate. The ridge would yield, while the suture would not. Fourteen years ago, he saw a gentleman, from the West Indies, wearing a lateral incisor, on a plate which run back to the second molar, but did not extend to the median line. It adhered very firmly; and he thought its success owing to the fact of its resting only on the soft parts. He would like to avoid clasps, but can't get prices to justify, as Dr. Smith has remarked.

Dr. RICHARDSON was favorable to the cavity plate. It does better at the start, and the patient is, therefore, better pleased. He thought we should endeavor to avoid clasps. 1st. In persons under eighteen or twenty years of age; 2d. In those who have soft teeth, even if adults; 3d. When the remaining teeth are all well formed and crowded. He would hate to separate such to insert clasps. He said it was a misfortune that, in using clasps, we must put them on the best teeth. He always did it under protest.

No. 5, having been referred to a standing committee, was not discussed. W.

